# Case report: Misdiagnosis of primary mucinous cystadenoma of the testicle by ultrasound

**DOI:** 10.3389/fonc.2023.1206697

**Published:** 2023-09-05

**Authors:** Linlin Zhang, Jianyuan Xuan, Manxi Li, Mei Zhang, Yu Song, Ziang Pan, Bo Fan, Lin Lu, Hongyan Zhou, Yang Li

**Affiliations:** ^1^ Department of Ultrasound, The Second Affiliated Hospital of Dalian Medical University, Dalian, China; ^2^ Department of Pathology, The Second Affiliated Hospital of Dalian Medical University, Dalian, China; ^3^ Department of Urology, The Second Affiliated Hospital of Dalian Medical University, Dalian, China

**Keywords:** testicle, cystadenoma, mucinous, ultrasound, diagnostic errors

## Abstract

Testicular mucinous cystadenoma is a rare benign testicular tumor with the characteristics of being potentially malignant and showing atypical clinical symptoms; this article reports a case of a primary testicular mucinous cystadenoma misdiagnosed as testicular teratoma by ultrasound. A 69-year-old man was admitted to the hospital because of a 1-year history of left-sided testicular enlargement with scrotal swelling and no obvious abnormalities on laboratory tests. Ultrasound examination revealed solid-mass lesions in the left testicle, suggesting a high probability of teratoma, and contrast-enhanced magnetic resonance imaging (MRI) examination suggested an increased possibility of epidermoid cysts. Contrast-enhanced computed tomography (CT) and contrast-enhanced MRI of the preoperative abdomen and pelvis showed no other primary adenocarcinoma. Doctors performed radical resection of the left testicle. The postoperative pathological diagnosis was mucinous cystadenoma, and calcification as well as partially mildly atypical epithelial cells were discovered. Furthermore, there were no neoplastic lesions in the epididymis or spermatic cord. No metastatic lesions were found after 6 months postoperative follow-up, and the patient’s prognosis remains good.

## 1 Introduction

Primary testicular mucinous cystadenoma is a rare ovarian-type tumor. There is little literature reporting the imaging features of this disease, and testicular mucinous cystadenoma is often undiagnosed or misdiagnosed. This article retrospectively analyzes a case of testicular mucinous cystadenoma misdiagnosed by ultrasound, hoping to improve sonographers’ understanding of testicular mucinous cystadenoma and further improve the ability to diagnose this disease.

## 2 Case presentation

**Figure 1:**
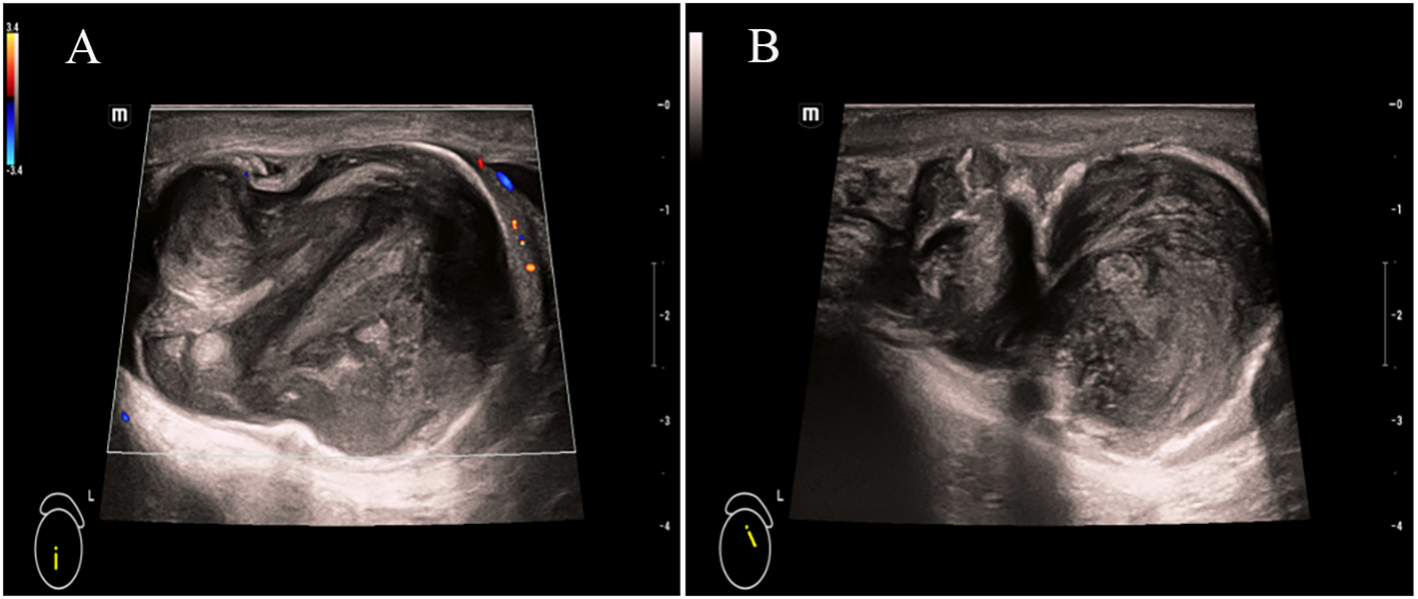
Ultrasonography of the scrotum showed a mass with disordered echo that occupied most of the left testis, which was compressed into a crescent-shaped shape and showed a small amount of blood flow signal in the testicular parenchyma. **(A)** Arcuate and coarse calcifications were detected in some areas **(B)**.

**Figure 2:**
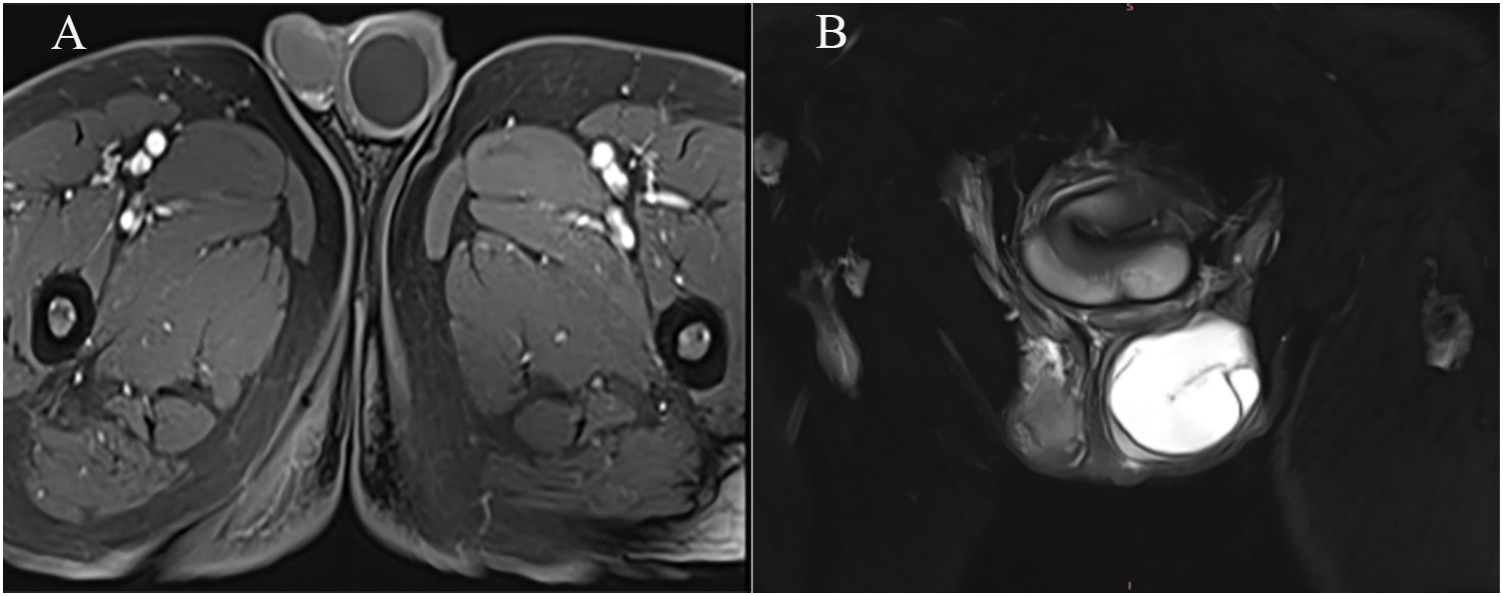
TIWI+C showed a round-like low signal, and mild reinforcement was visible at the edges. **(A)** T2WI showed that the left testis was enlarged, high signal intensity opacities were visible, and a linear low signal intensity was visible at the margins and internally **(B)**.

**Figure 3:**
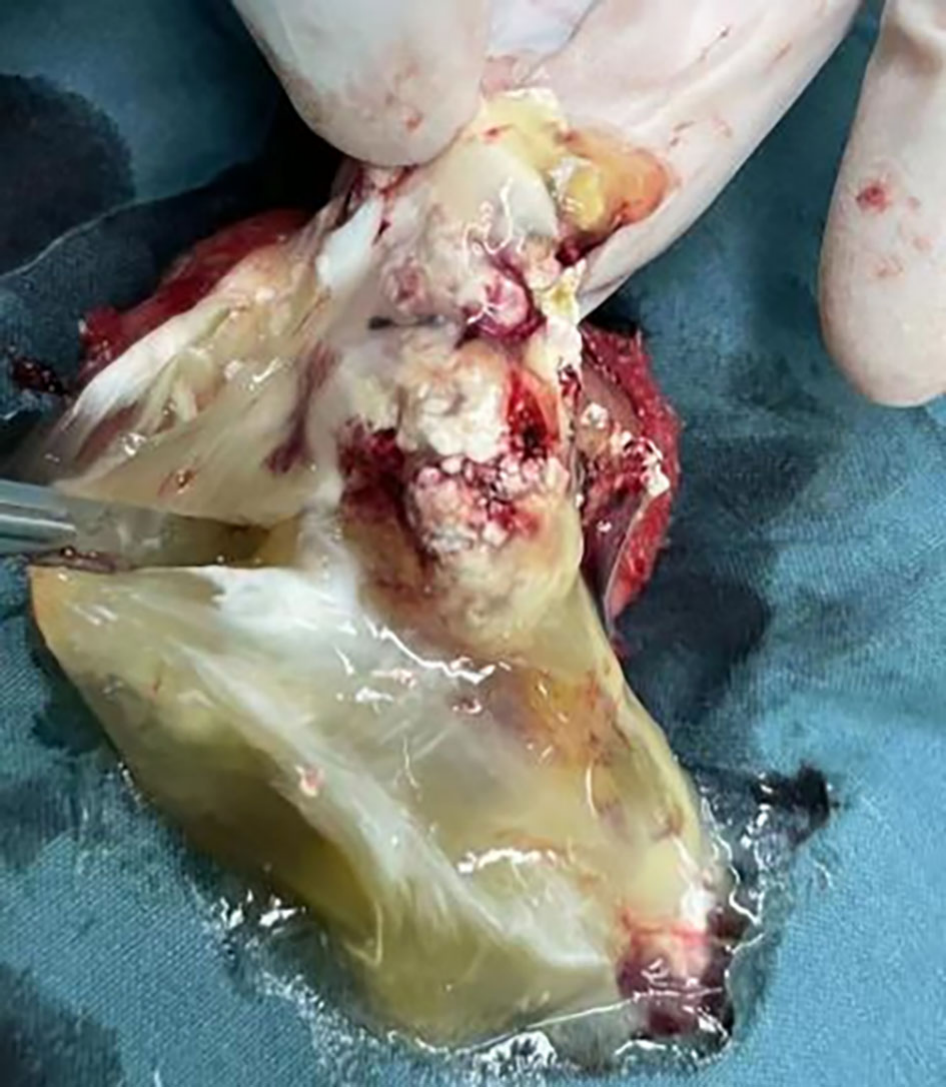
The general pathology showed that the partial incision of the incision surface was solid, the solid area was gray-yellow and soft, the inner wall of the cystic capsule was slightly rough, and the local gray-yellow and gray-white granular areas were full of jelly-like matter.

**Figure 4:**
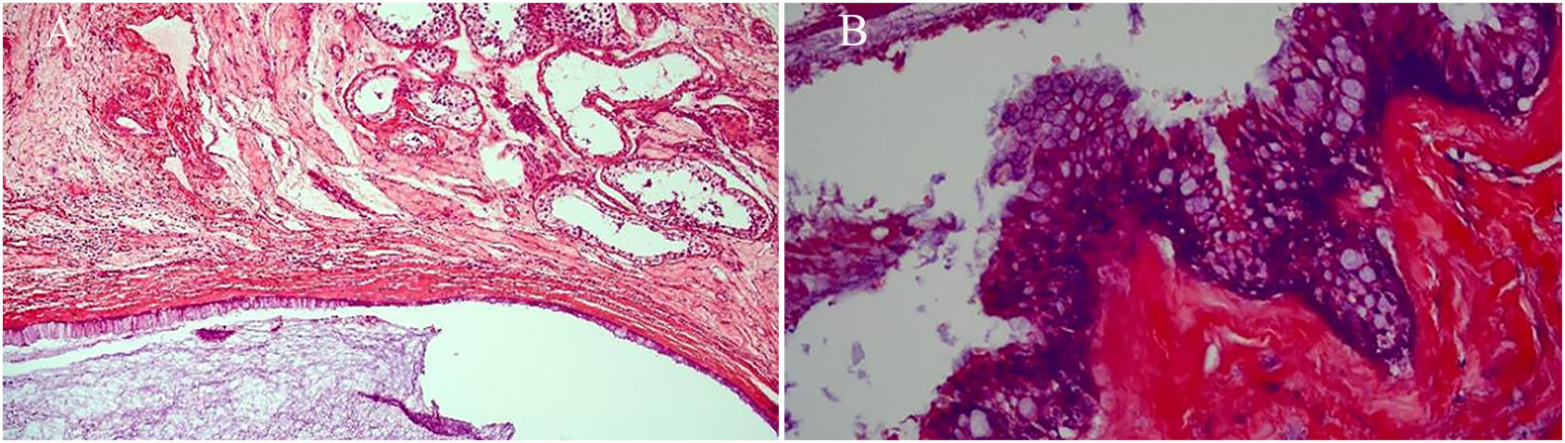
Microscopically, there were multiple cystic glands and fibrotic interstitium, the cyst wall was lined with columnar mucus epithelium, and the local mucous epithelium had mild atypia and no interstitial infiltrates. (**A**: HE×100, **B**: HE×400).

The patient, a 69-year-old male, was admitted to the hospital due to a 1-year history of left-sided testicular enlargement with scrotal swelling, with no pain or other notable discomfort. He had no history of scrotal trauma and no family history or genetic history. Physical examination showed enlargement of the left testicle, no redness or rupture on the surface, normal skin temperature, and a palpable mass in the left testicle, which was tough and nontender. The markers related to testicular tumors, such as alpha-fetoprotein (AFP), β-human chorionic gonadotropin (β-HCG) and lactate dehydrogenase (LDH), were all within their normal ranges, and no obvious abnormalities were found in other laboratory tests. Ultrasound examination showed an enlarged left testicle with a hypoechoic mass that occupied almost the entire testicle. The left testicular parenchyma was compressed into the shape of a crescent, and a small amount of blood flow signal could be seen in the testicular parenchyma (**Figure 1A**), but no abnormalities were seen in the right testicle. The size of the mass was 4.2×3.6×3.0 cm, and it had a clear border, disordered internal echo, and arcuate and coarse calcifications visible in some areas (**Figure 1B**). There was no effusion in the left and right testicular vaginal tunica cavity, and there were no abnormalities in the bilateral epididymis and spermatic cord. Ultrasound examination suggested solid mass lesions in the left testicle and a high probability of teratoma. MRI showed an enlarged left testis, visible round T1WI low signal and high signal on T2WI, visible linear low signal at the edge and internally, slightly higher signal on DWI, and high signal on DC MRI; the area measured approximately 3.8×4.0×4.5 cm, with mild enhancement along the edge seen on contrast-enhanced scanning (**Figures 2A, B**). The scanning range of the pelvic cavity and bilateral inguinal area showed no enlarged lymph nodes, and the diagnosis of the abnormal focus in the left testis considered the possibility of an epidermoid cyst. The patient underwent radical resection of the left testicle, and the postoperative pathology showed that the size of the testicle was 4.0×3.0×2.5 cm. The general specimen was partially dissected, and the incision surface was solid. The solid area measured 4.0×3.0×0.7 cm and was gray-yellow and soft, and the cystic area measured 3.0×3.5×2.0 cm. The inner wall of the capsule was slightly rough with local gray-yellow and gray-white granules, which were filled with jelly-like matter (**Figure 3**). There were no neoplastic lesions in the epididymis or spermatic cord. Microscopically, multiple cystic glands and fibrotic interstitium were seen, the cyst wall was lined with columnar mucus epithelium, and the local mucous epithelium had mild atypia with no interstitial infiltrates (**Figures 4A, B**). Follow-up 6 months later showed no tumor recurrence or metastasis.

## 3 Discussion

According to the 2022 World Health Organization classification of tumors of the urinary system and male genital organs, testicular mucinous cystadenoma belongs to the ovarian-type tumors of the collecting ducts and rete testis of the testicular adnexal tumors (1). Ovarian-type tumors of the collecting ducts and rete testis tumors include serous, mucinous, endometrioid, clear cell and Brenner tumors (**Table 1**). Serous subtypes are the most common, and mucinous subtypes are extremely rare (1, 2). Similar to ovarian tumors, testicular tumors’ biological behavior can be classified as benign, borderline and malignant, and testicular mucinous cystadenoma is a rare benign testicular tumor with potential malignancy (1–3). To date, a total of 31 cases of testicular or paratesticular ovarian mucinous tumors have been reported, including 9 cases of mucinous cystadenoma, 15 cases of borderline mucinous cystadenoma, and 7 cases of mucinous cystadenocarcinoma (**Table 2**) (2–25). Most patients presented with painless scrotal enlargement, only 4 patients had mild scrotal pain (4, 9, 13, 16), patients often had unilateral onset, and only 1 patient was bilateral (21). The median age of the patients was 55 years (11-71 years), and the age of 55 years and older accounted for 22.2% (2/9), 60.0% (11/15) and 85.7% (6/7) of the testicular or paratesticular mucinous cystadenomas, borderline mucinous cystadenomas, and mucinous cystadenocarcinomas, respectively. Among the patients with testicular or paratesticular mucinous cystadenoma and borderline mucinous cystadenoma, 4 patients were lost to follow-up, 17 patients had no local recurrence or metastasis, 1 patient had metastasis, and 2 patients died of other diseases. Among patients with testicular or paratesticular mucinous cystadenocarcinoma, 5 had no recurrence or metastasis, 1 had metastasis, and 1 died from the disease. At present, primary testicular mucinous cystadenoma is often missed during diagnosis, delayed in diagnosis or misdiagnosed due to a lack of relevant literature reporting its imaging features, the lack of typical symptoms in the early stage, and psychological reasons of the patients.

**Table 1:** Ovarian-type tumours of the collecting ducts and rete testis.

Type	Classification
Serous	Serous cystadenoma
	Serous borderline tumour
	Serous cystadenocarcinoma
Mucinous	Mucinous cystadenoma
	Mucinous borderline tumour
	Mucinous cystadenocarcinoma
Endometrioid	Endometrioid tumour, borderline
	Endometrioid adenocarcinoma
Clear cell type	Clear cell adenocarcinoma
Brenner type	Brenner tumour

**Table 2:** Summary of reported primary testicles or paratesticular mucinous tumors.

Type	Location	Age(y)	Follow up	Prognosis	Reference
Mucinous Cystadenoma	Paratesticular	11	14y	No disease recurrence	(4)
	Paratesticular	18	1.5y	No disease recurrence	(5)
	Paratesticular	54	1.8y	No disease recurrence	(2)
	Testicular	35	8m	No disease recurrence	(6)
	Testicular	39	1y	No disease recurrence	(7)
	Testicular	43	2.5y	No disease recurrence	(8)
	Testicular	54	3m	No disease recurrence	(9)
	Testicular	55	5m	No disease recurrence	(10)
	Testicular	61	unknown	unknown	(11)
Borderline mucinous tumor	Paratesticular	42	unknown	unknown	(12)
	Paratesticular	54	unknown	unknown	(13)
	Paratesticular	65	7y	No disease recurrence	(2)
	Paratesticular	66	10y	Dead of other causes	(14)
	Paratesticular	68	12y	Dead of other causes	(2)
	Testicular	21	6m	Metastasis	(15)
	Testicular	44	8y	No disease recurrence	(2)
	Testicular	45	unknown	unknown	(16)
	Testicular	52	4y	No disease recurrence	(17)
	Testicular	55	3.7y	No disease recurrence	(18)
	Testicular	59	4y	No disease recurrence	(2)
	Testicular	60	4y	No disease recurrence	(19)
	Testicular	64	2y	No disease recurrence	(2)
	Testicular	69	4y	No disease recurrence	(2)
	Testicular	69	3y	No disease recurrence	(20)
Mucinous carcinoma	Paratesticular	69	2m	Dead of disease	(2)
	Testicular/epididymus	59	2y	No disease recurrence	(21)
	Testicular	44	2y	No disease recurrence	(22)
	Testicular	55	1y	No disease recurrence	(23)
	Testicular	60	2y	No disease recurrence	(24)
	Testicular	67	2y	Metastasis	(25)
	Testicular	71	1y	No disease recurrence	(3)

To date, the origin of the disease remains unknown, but the possibilities are as follows: mucinous metaplasia of the mesothelium of the sheath (2, 8), residue of Müllerian ducts in the contents of the appendix, testis, or outer scrotum of the testis in humans (5, 8), and unilateral differentiation of teratoma cells (6, 10, 11). Most of the patients reported in the literature are older and the tumors were much more advanced than germ cell tumors. In addition, most patients presented with painless scrotal enlargement or a unilateral testicular mass, and some patients also had hydrocele vessels. Furthermore, most patients underwent mucus extravasation and relevant fibrosis and calcification (2, 3, 6, 8, 11, 17, 18), and serum tumor markers such as AFP, β-HCG, and LDH, which may be associated with testicular tumors, were negative (6, 8–11, 18). The gross pathology and microscopic features of the tumor are similar to those of ovarian mucinous tumors, and most have intestinal cell-like features and, occasionally, Müllerian cytolike features (2, 3, 5–7). The cyst tissue consists of mucous epithelial cells with high columnar, endocervix-like cells without atypical nuclei (2, 8, 10, 18). Testicular mucinous cystic tumors have been reported to be likely positive for CK7 and CK20 or positive for CK20 and negative for CK7 (2, 3, 7, 9–11). In addition, Kim et al. found that CDX2 may also be expressed in testicular mucinous cystadenoma (11).

Because testicular mucinous cystadenomas are rare, this case highlights the ultrasound features of the disease and compares the ultrasound findings with other diseases, hoping to improve the sonographer’s awareness and diagnosis of the disease and, more importantly, increase urologists’ vigilance for the disease and exclude the source of metastasis to ensure optimal outcomes. Ultrasonography is a commonly used imaging test for diagnosing testicular tumors and is particularly important in determining the size of the mass and its relationship to the ipsilateral testis. Ultrasound examination mostly shows an enlarged testicle with a hypoechoic mass that occupied almost the entire testicle, and the mass had a clear border, cluttered internal echo, arcuate and coarse calcifications or liquid anechoic zone visible in some areas, and no or a small amount of blood flow signal (5, 7, 11, 17). Primary testicular mucinous cystadenomas are usually differentiated from testicular teratomas and metastatic testicular mucinous cystadenomas due to the lack of specific ultrasound findings. Testicular teratomas are the most common testicular tumor in children, and ultrasonography often reveals a well-circumscribed cystic or mixed lesion, and a small amount of blood flow signal may be seen. Mature teratomas are cystic lesions with septal and fatty echoes visible, while immature teratomas are mixed lesions with capsules and solid areas, and the lesions often have cystic degeneration, bleeding or calcification (26–28). Compared with primary testicular mucinous tumors, metastatic mucinous tumors from the digestive system are reported to be more common and have a very poor prognosis, so clinical doctors can exclude metastatic mucinous tumors through a variety of examination methods, such as CT, MRI, gastrointestinal endoscopy, and tumor markers (2, 17, 29, 30). In this case, the diagnosis of the testicular mass of this patient excluded the possibility of tumors of other tissue origins, particularly digestive origins, by imaging examination. Therefore, the patient was considered to have a diagnosis of primary mucinous cystadenoma of the testicle. In addition, the combination of conventional ultrasound with contrast ultrasonography and elastography can help with the diagnosis, and malignancy can be suspected when contrast ultrasonography of the diseased tissue shows excessive hyperenhancement or when ultrasound elastography suggests a “hard” texture (28). Although the mass was diagnosed as cystic by MRI, we could not diagnose it as cystic or mixed by ultrasound because the liquid contained in the mass was thick and the probe did not feel suspended after pressurization, and it was confirmed to be a jelly-like substance by pathology. The mass echo was cluttered and contained calcifications, so we mistakenly judged it as a solid mass and then misdiagnosed it as a malignant tumor, that is, teratoma. At the same time, the lack of ultrasound elasticity and contrast-enhanced ultrasound had a certain impact on the diagnosis of the disease.

Current experience in the treatment of primary testicular mucinous cystadenoma is limited, and there is no standardized staging protocol or treatment. Therefore, radical orchiectomy is the best treatment option when the ultrasound findings are suspicious or when the nature of the testicular lesions is difficult to determine preoperatively. Compared with malignant tumors, primary testicular mucinous cystadenomas have a lower metastasis rate and recurrence rate and a significantly better prognosis (**Table 2**) (18). While favorable clinical outcomes have been reported in most previous cases, we should consider that this tumor has the potential to be malignant. It has been reported in the literature that a 21-year-old patient with borderline testicular mucinous cystadenoma was misdiagnosed with testicular mucinous cystadenoma after surgery, and the incorrect pathological results and no obvious symptoms led to follow-up examination being neglected. Multiple metastases occurred 6 months after surgery, and the prognosis of the patient was poor. Therefore, we recommend close follow-up of patients with this type of tumor. This patient in the current case did not show features of disease recurrence or metastasis when monitored by tumor marker levels, scrotal ultrasound, abdominal and pelvic CT, and gastrointestinal endoscopy. His postoperative recovery was very smooth and his prognosis was good.

## 4 Conclusion

Primary testicular mucinous cystadenoma is a rare ovarian-type collecting duct and testicular mesh tumor that is usually benign with rare malignant manifestations. Testicular mucinous cystadenoma disease should be considered in middle-aged and elderly men with painless scrotal swelling or a unilateral testicular mass and an ultrasound examination that shows that the mass has clear boundaries, disorganized internal echoes, calcification in some areas seen, and no or small amount of blood flow signal. Moreover, abdominal and pelvic CT and gastrointestinal endoscopy are required to exclude metastatic mucinous tumors. Pathological examination is the gold standard for diagnosing the disease. The currently recommended treatment regimen is radical orchiectomy with close follow-up.

## Data availability statement

The original contributions presented in the study are included in the article/supplementary material. Further inquiries can be directed to the corresponding authors.

## Ethics statement

Written informed consent was obtained from the individual(s) for the publication of any potentially identifiable images or data included in this article.

## Author contributions

YL and HZ designed and revised the manuscript. LZ drafted the manuscript. JX, ML and MZ helped to revise the manuscript. YS, ZP, BF and LL provided guidance and the proofreading of the article. All authors contributed to the article and approved the submitted version.
